# Viral etiology of hospitalized acute lower respiratory infections in children under 5 years of age – a systematic review and meta-analysis

**DOI:** 10.3325/cmj.2013.54.122

**Published:** 2013-04

**Authors:** Ivana Lukšić, Patrick K Kearns, Fiona Scott, Igor Rudan, Harry Campbell, Harish Nair

**Affiliations:** 1 Institute of Public Health ''Dr. Andrija Štampar,'' Department of Microbiology, Zagreb, Croatia; 2 Centre for Population Health Sciences, The University of Edinburgh Medical School, Edinburgh, Scotland, UK; 3 Public Health Foundation of India, New Delhi, India; * These authors contributed equally

## Abstract

**Aim:**

To estimate the proportional contribution of influenza viruses (IV), parainfluenza viruses (PIV), adenoviruses (AV), and coronaviruses (CV) to the burden of severe acute lower respiratory infections (ALRI).

**Methods:**

The review of the literature followed PRISMA guidelines. We included studies of hospitalized children aged 0-4 years with confirmed ALRI published between 1995 and 2011. A total of 51 studies were included in the final review, comprising 56 091 hospitalized ALRI episodes.

**Results:**

IV was detected in 3.0% (2.2%-4.0%) of all hospitalized ALRI cases, PIV in 2.7% (1.9%-3.7%), and AV in 5.8% (3.4%-9.1%). CV are technically difficult to culture, and they were detected in 4.8% of all hospitalized ALRI patients in one study. When respiratory syncytial virus (RSV) and less common viruses were included, at least one virus was detected in 50.4% (40.0%-60.7%) of all hospitalized severe ALRI episodes. Moreover, 21.9% (17.7%-26.4%) of these viral ALRI were mixed, including more than one viral pathogen. Among all severe ALRI with confirmed viral etiology, IV accounted for 7.0% (5.5%-8.7%), PIV for 5.8% (4.1%-7.7%), and AV for 8.8% (5.3%-13.0%). CV was found in 10.6% of virus-positive pneumonia patients in one study.

**Conclusions:**

This article provides the most comprehensive analysis of the contribution of four viral causes to severe ALRI to date. Our results can be used in further cost-effectiveness analyses of vaccine development and implementation for a number of respiratory viruses.

Acute lower respiratory tract infections (ALRI) are the leading cause of global mortality in children under five years of age (1,2). Studies of pre-school children from developed and developing countries alike suggest that the majority of respiratory infections generally have viral etiology (2-4). Clinically, ALRIs can be divided into pneumonias and bronchiolitis (5,6). Differentiating those two conditions can be particularly difficult in younger children, who typically exhibit less specific clinical symptoms (3,7-9). In high-income countries (HIC), pneumonia rarely causes deaths in children (10), although it continues to be a major cause of morbidity and poses a significant economic burden (11). Bronchiolitis is characterized by a distressing pattern of symptoms: low-grade/absent fever progressing to cough, coryza, tachypnoea, hyperinflation, chest retraction, and widespread crackles or wheezes (12). Bronchiolitis deaths are very rare in HIC (13,14), but children are at increased risk of recurrent wheezing and the data on mortality in low and middle income countries (LMIC) are scarce (15).

Etiology of severe ALRI episodes is not well understood: limited contribution of the three major pathogens (*S. pneumoniae*, *H. influenza,* and respiratory syncytial virus) is established, but the role of other viruses has not been explored. The importance of viruses as major causes of ALRI is becoming increasingly apparent because the sensitivity of detection techniques has greatly improved and new molecular tests increasingly replace conventional methods. The use of polymerase-chain reaction (PCR) now allows identification of viruses that have previously been difficult or impossible to culture. In the past decade, numerous novel respiratory viruses that can cause ALRI have been discovered, and new diagnostic methods for the use in high and low-resource settings alike are continuously evolving (3,16-20). It seems that the conventional diagnostic methods have systematically underestimated the role of viruses as causal pathogens in ALRI (3), and also that viruses are capable of causing severe, life-threatening ALRI (3). The emergence of the severe acute respiratory syndrome (SARS), caused by a novel coronavirus, and the avian influenza type A (H5N1) outbreak are good recent examples (16,20).

Impressive progress has been made in the last decade in increasing the global availability of vaccines against the main bacterial causes of ALRI – *S. pneumoniae* and *H. influenzae type B* – leading to marked reductions in both hospitalizations and deaths (21,22). This will lead to increased focus on viral causes and their prevention and management. Strains of influenza type A and B viruses can be life threatening (3), although infection in the majority of young children is vaccine-preventable (23,24). Parainfluenza viruses (PIV) are the most common cause of croup in young children, with PIV1 and PIV3 also being the causes of severe bronchiolitis and pneumonia (3,4,25), but there are currently no licensed PIV vaccines. Adenoviruses (AV) have long been recognized as pathogens of the lower respiratory tract that can be associated with severe or lethal lower respiratory tract infection (3,26,27) or bronchiolitis obliterans (28-31). Coronaviruses (CV) cause common cold and have been historically thought to be a very rare cause of ALRI (32), despite the fact that they sporadically caused catastrophic disease in livestock (33). The SARS-CV outbreak in 2003, which was a highly virulent zoonosis capable of human-to-human transmission, renewed the interest in CV as human pathogens (32). This led to a discovery of two previously unrecognized CVs as causes of ALRI (16,17).

This study analyzed the available information on the role of four viruses (IV, PIV, AV, and CV), all of which have been historically considered to be relatively uncommon causes of severe ALRI in hospitalized cases. Our study did not assess the role of common causes – RSV, S. pneumonia, and H. influenzae – because their roles have already been systematically characterized and well-established (34). We are not aware of any systematic analyses of the global prevalence of viruses in severe childhood ALRI. We aimed to assess the proportion of cases of severe ALRI with a viral etiology and explore the contribution of mixed viral infections and separate contributions of IV, PIV, AV, and CV to severe ALRI in children under five years of age.

## Methods

This systematic review was carried out using the PRISMA and MOOSE protocols (35-37). These protocols have been developed to ensure standardized and replicable approach to systematic review of the available evidence on the burden of specific health problems, the role of risk factors, or the effectiveness of available health interventions, and the unified reporting of the findings.

### Literature search and inclusion criteria

A systematic literature review was performed using the search terms detailed in Supplementary online material(supplementary material). This was supplemented by hand searching of key online journals and reference lists of selected papers. The search included the following databases: Medline, EMBASE, CINAHL, Global Health Library, WHOLIS, LILACS, IndMed, AIM, SciELO, IMEMR, IMSEAR, WPRIM, and SIGLE (gray literature).

All studies included in the analysis reported on inpatients aged 0-4 years with a clinical diagnosis of community-acquired ALRI, bronchiolitis, or pneumonia. Investigation of viral etiology was a requirement and the participants needed to be free of co-morbid conditions. Children admitted to emergency departments were excluded, and so were intensive care patients wherever data was not reported for all other inpatients in the hospital, to avoid potential bias. We included studies conducted between 1995 and 2011 with a continuous study period of one year (or multiples of one year), to avoid effects of seasonality. Studies that relied solely on serology for diagnosis were excluded, because this method could not reliably differentiate acute from past infections (38,39). Studies that were conducted during an epidemic or pandemic outbreak were also excluded.

### Study selection and data extraction

**Figure 1:**
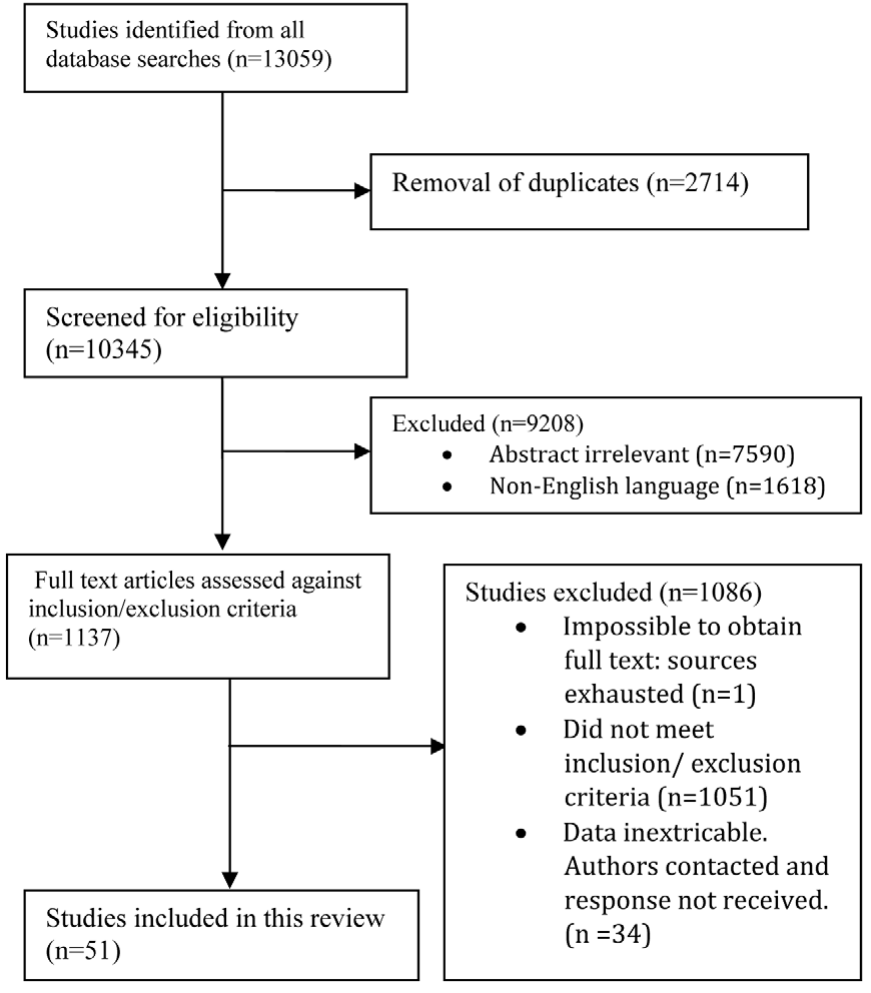
Details of the systematic review and study selection process.

Study selection was performed following the removal of duplicates. Authors were contacted by email in cases when study data were not published in an extractable form, to collect further details. Data were extracted for study location, period of study, sample, diagnostic assay, clinical diagnosis, age range and median age of study population, potential etiological agents investigated, proportion of patients in whom no etiological diagnosis was found, viruses and bacteria detected, and age breakdown of patients by diagnosis where available (Figure 1).

### Assessment of bias within studies

During the process of data extraction, information was drawn from each study on possible sources of bias that could affect the results, such as:

• Respiratory sample used (as there is no “gold standard;” samples from lower respiratory tract are preferable, but they require invasive procedures and are difficult to obtain without contamination from the upper airway; because of this, most studies consider nasopharyngeal aspirates for viral detection as acceptable, although acknowledging limitations. Viruses detected in the upper airway of a patient with ALRI are not necessarily pathogens of the lower respiratory tract);

• HIV co-infection (as this is known to increase the susceptibility to ALRI and the rate of atypical infection) (40);

• Viral detection technique and timing (as there is large variability in the sensitivity of different techniques; viral culture can only reliably be used within 2 days of onset of acute rhinorrhea, when viable virus shedding is at its peak (41); PCR can be used much later, because it does not require viable viruses in the sample, offering much improved sensitivity, but also greatly increased rates of detection of benign co-infections).

### Summary measures

Proportional contributions of IV, PIV, AV, and CV to severe ALRI and associated confidence intervals were derived through meta-analysis using StatsDirect software package (StatsDirect Ltd, Academic version 2.7.9., Cheshire, UK). Due to large variation in methodology and patient demographics between studies, random effects models were used in all analyses, as proposed by DerSimonian and Laird (42). Heterogeneity and bias analyses were also performed for all meta-analyses. All presented results were shown to be free of publication bias, as demonstrated using funnel plots and analysis methods proposed by Begg (43), Egger (44), and Harbold (45). This triple approach represents robust protection from the sources of bias.

## Results

**Figure 2:**
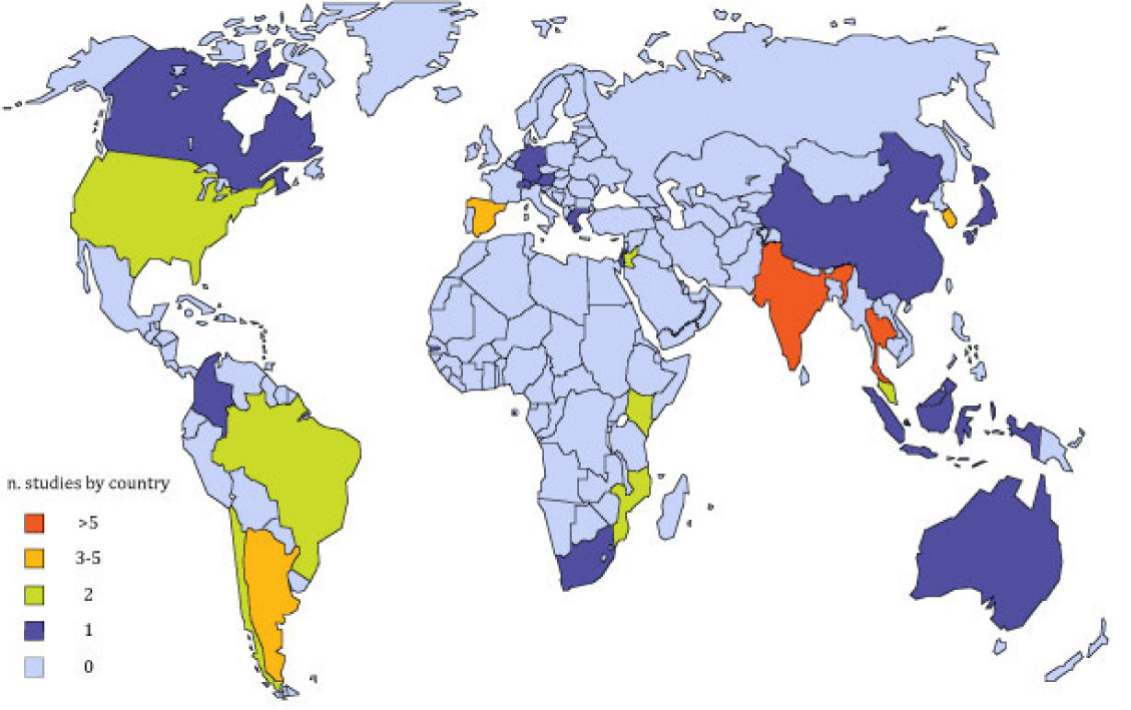
Geographic distribution of studies included in this review (N = 51).

**Figure 3:**
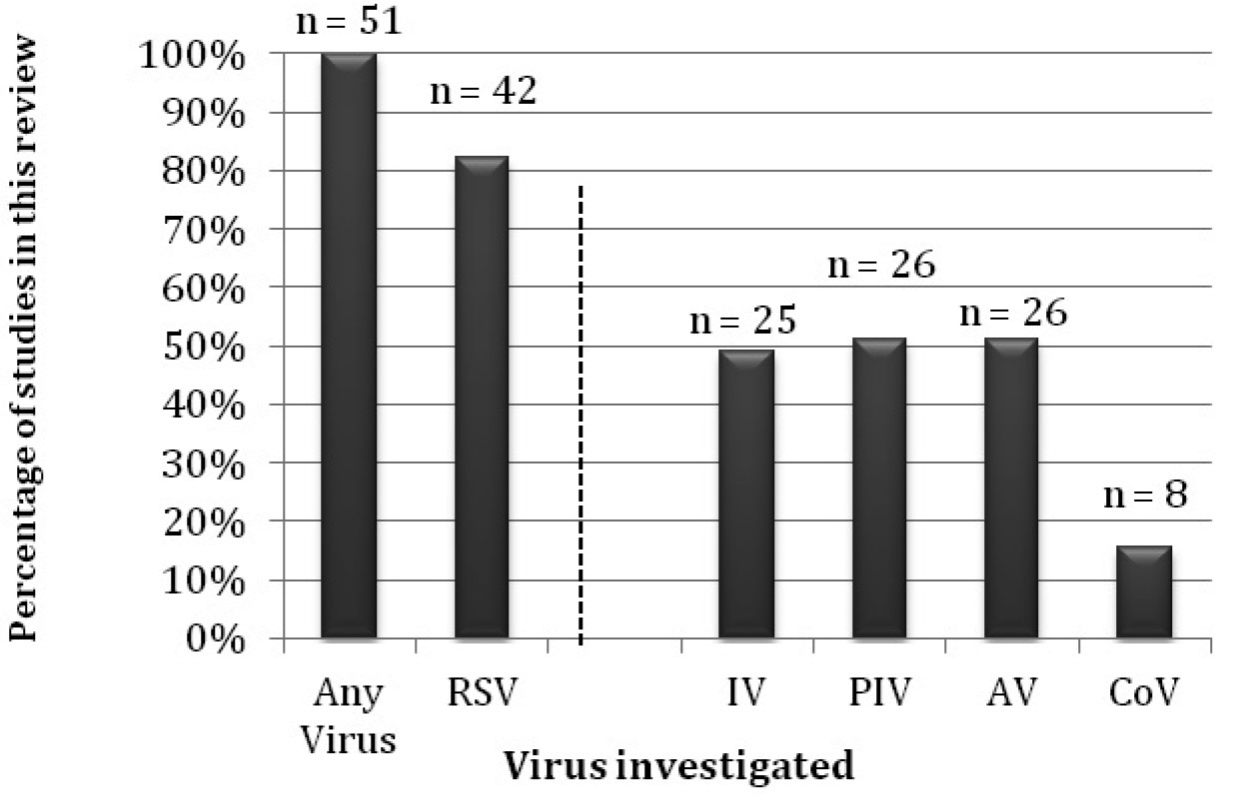
Proportion of studies retained for the final analyses that investigated individual viruses: approximately half investigated influenza virus (IV), parainfluenza virus (PIV), and/or adenovirus (AV), with no studies on coronavirus (CV) before the 2003 SARS-CV outbreak. Twenty studies only described one viral agent (11 of these respiratory syncytial virus, RSV).

Fifty one studies meeting the inclusion criteria were included in this review, including 56 091 episodes of severe hospitalized ALRI. Figure 2 presents geographical distribution of the retained studies, Figure 3 shows proportion of studies investigating different viruses (any virus, RSV, IV, PIV, AV, CV), while Table 1 presents their basic characteristics in terms of case definition, sample size, period of study, and diagnostic methods used (15,40,46-94). Only four studies investigated children hospitalized with ALRI for both bacterial and viral etiology (59,64,69,74) and only six studies reported HIV co-infection as their exclusion criteria (15,58,59,63,64,86). A total of 19 studies were from high-income countries (95), investigating on average 6.5 viruses per study, while studies in LMIC investigated 2.7 viruses (unpaired *t* test: *P* = 0.002). It seems likely that this difference reflects the fact that more tests are typically used in establishing diagnosis in high-income settings, without certainty over the causal role of all identified viral pathogens, and this may introduce systematic bias and heterogeneity between studies in HIC and LMIC.

**Table 1:** A description of basic characteristics of the included studies (15,40,46-94)

Author and reference number	Year	Country	Case def.	Cases (n)	Period of study	Age range (months)	Sample	Diagnostic assay	Viruses tested (n)
**Aberle, J.H., et al. (****46****)**	2005	Austria^†^	LRTI^‡^	772	Oct 2000 - July 2004	<12	NPA	PCR	5
**Al-Toum, R., et al. (****47****)**	2009	Jordan	LRTI	141	Sep 2002 - Mar 2004	<24	NPA	Culture	1
**Avendano, L.F., et al. (****48****)**	2003	Chile	LRTI	4618	Jan 1989 - Dec 2000	<24	NPA	IFA	1
**Banerji, A., et al. (****49****)**	2009	Canada^†^	LRTI	121	Jan 2002 - Mar 2003	<24	NPA	IFA and PCR	20
**Bdour, S., et al. (****50****)**	2001	Jordan	LRTI	271	Jan 1997 - May 1999	<24	NPW	IFA	1
**Bedoya, V.I., et al. (****51****)**	1996	Colombia	LRTI	103	Apr 1994 - Apr 1995	<12	NPW	IFA	1
**Bharaj, P., et al. (****52****)**	2010	India	LRTI	181	Apr 2005 - Mar 2007	<62	NPA	PCR	1
**Bharaj, P., et al. (****53****)**	2009	India	LRTI	135	Apr 2005 - Mar 2007	<72	NPA	PCR	20
**Bolisetty, S., et al. (****54****)**	2005	Australia^†^	AB	167	Jan 2000 - Dec 2000	<24	NPA	EIA and Culture	4
**Calvo, C., et al. (****55****)**	2010	Spain^†^	AB	318	Sep 2005 - Aug 2008	<12	NPA	PCR	16
**Canducci, F., et al. (****56****)**	2008	Spain^†^	LRTI	230	Oct 2004 - Sep 2006	<24	NPA	PCR	4
**Carballal, G., et al. (****57****)**	2000	Argentina	LRTI	1304	Jan 1990 - Dec 1996	<24	NPA	IFA	1
**Carballal, G., et al. (****58****)**	2001	Argentina	LRTI	1234	Apr 1993 - Dec 1994	<60	NPA	IFA	4
Cevey-Macherel, M., et al. * (**59****)**	2009	Switzerland^†^	CAP	99	Mar 2003 - Dec 2005	<60	NPA	PCR	7
**Chakravarti, A., et al. (****60****)**	1995	India	LRTI	45	Jul 1990 - Jun 1991	<24	NPA	EIA	1
**Chan, K.B., et al. (****61****)**	1999	Malaysia	LRTI	5691	Jan 1982 - Dec 1997	<24	NPA	IFA and Culture	4
**Charanjit, K., et al. (****62****)**	2010	India	AB	245	Jan 2007 - Dec 2007	<12	NPA/NPW	PCR, EIA and Culture	6
**Choi, E., et al. (****63****)**	2006	Rep. Korea^†^	LRTI	515	Sep 2000 - Aug 2005	<60	NPA	PCR	11
Chong, C.Y., et al. * (**64****)**	1997	Singapore^†^	LRTI	333	May 1994 - Apr 1995	<60	NPA	Not declared	4
**Chung, T., et al. (****65****)**	2007	Rep. Korea^†^	LRTI	233	Jul 2004 - Jan 2006	<60	NPA	PCR + IFA	7
**Cifuentes, L., et al. (****15****)**	2003	Chile	AB	36	May 1999 - Aug 2000	<24	NPA	EIA	1
**Cilla, G., et al. (****66****)**	2009	Spain ^†^	LRTI	533	Jul 2004 - Jun 2007	<35	NPA	PCR	1
**Dare, R.K., et al. (****67****)**	2007	Thailand	CAP	510	Sep 2003-Aug 2005	<60	NPS	PCR and Culture	1
**Djelantik, I.G., et al. (****68****)**	2003	Indonesia	LRTI	2677	Jan 2000 - Dec 2001	<24	NPW	EIA	1
Ekalaksananan, T., et al. * (**69****)**	2001	Thailand	LRTI	62	Aug 1992 - Nov 1994	<60	NPA	EIA and Culture	2
**Fry, A.M., et al. (****70****)**	2011	Thailand	CAP	352	Sept 2003-Aug 2005	<60	NPS	PCR and Culture	1
**Fry, A.M., et al. (****71****)**	2007	Thailand	CAP	369	Sep 2004 - Aug 2005	<60	NPS	PCR	1
**Garcia, C.G., et al. (****72****)**	2010	USA^†^	AB	4285	Jan 2002 - Dec 2007	<23	NPA	IFA and Culture	5
Kabra, S.K., et al. * (**73****)**	2003	India	LRTI	95	Mar 1995 - Feb 1997	<60	NPA	Culture	4
**Kabra, S.K., et al. (****74****)**	2004	India	LRTI	200	Mar 1995 - Feb 1997	<60	NPA	Culture	4
**Kim, Y.K., et al. (****75****)**	2005	Rep. Korea^†^	LRTI	166	Aug 1997 - Mar 2000	<60	NPA	PCR	5
**Loscertales, M.P., et al. (****76****)**	2002	Mozambique	LRTI	1001	Oct 1998 - May 2000	<60	NPA	EIA	1
**Moodley, T., et al. (****40****)**	2010	S. Africa	AB	106	Jan 2006 - Dec 2007	<24	NPA	IFA	6
**Moriyama, Y., et al. (****77****)**	2010	Japan^†^	LRTI	402	April 2007-July 2009	<24	NPS	PCR	6
**Nascimento-Carvalho, C.M., et al. (****78****)**	2011	Brazil	CAP	268	Sep 2003 - May 2005	<60	NPA	PCR	1
**Nokes, J., et al. (****79****)**	2008	Kenya	LRTI	223	Jan 2002 - Feb 2005	<30	NPA/NPW	IFA	1
**Nokes, J., et al. (****80****)**	2009	Kenya	CAP	6026	Jan 2002 - Dec 2007	<60	NPA/NPW	IFA	1
**O'Callaghan-Gordo, C., et al. (****81****)**	2011	Mozambique	CAP	807	Sep 2006 - Sep 2007	<60	NPA	PCR	7
**Oliveira, D.B., et al. (****82****)**	2009	Brazil	LRTI	226	Jan 2003 -Dec 2003	<60	NPA/NPS	PCR	2
**Samransamruajkit, R., et al. (****83****)**	2008	Thailand	CAP	239	Mar 2006-Feb 2007	<60	NPA	PCR	1
**Singleton, R.J., et al. (****84****)**	2010	USA^†^	LRTI	424	Oct 2005 - Sep 2007	<36	NPS/NPW	PCR	4
**Teeratakulpisarn, J., et al. (****85****)**	2007	Thailand	AB	170	Apr 2002 - Aug 2004	<24	NPA	PCR	2
**Videla, C., et al. (****86****)**	1998	Argentina	LRTI	158	May 1991-Dec 1992	<60	NPA	IFA	2
**Viegas, M., et al. (****87****)**	2004	Argentina	LRTI	18561	Jan 1998 - Dec 2002	<24	NPA	IFA	4
**Weber, M.W., et al. (****88****)**	2002	Gambia	LRTI	2252	Oct 1993 - Dec 1997	<60	NPA	IFA	1
**Weigl, J.A., et al. (****89****)**	2005	Germany^†^	CAP	187	Jul 1996 - Jun 2000	<60	NPA	PCR	5
**Wolf, D.G., et al. (****90****)**	2006	Israel^†^	CAP	88	Nov 2001 - Oct 2002	<60	NPW	IFA and PCR	3
**Xepapadaki, et al. (****91****)**	2004	Greece ^†^	AB	56	Oct 1999- Sep 2000	<24	NPW	PCR	5
**Xiang, Z., et al. (****92****)**	2010	China	CAP	384	Apr 2007-Mar 2008	<60	NPA	PCR	1
**Yin, C.C., et al. (****93****)**	2003	Singapore^†^	LRTI	1011	Aug 1998-Jul 1999	<60	NPA	IFA	4
**Yoo, S.J., et al. (****94****)**	2007	Rep. Korea^†^	LRTI	158	Jan 2004 - Dec 2004	<60	NPA	PCR and IFA	7

*Studies that investigate viral and non-viral etiological agents.

†High income country, as classified by the global burden of disease (GBD21), excluding studies of indigenous groups.

‡Abbreviations: CAP – Community acquired pneumonia; AB – acute bronchiolitis; LRTI – lower respiratory tract infection (or equivalent); NPA – nasopharyngeal aspirate; NPS – nasopharyngeal swab; NPW – nasopharyngeal wash; PCR – polymerase chain reaction; IFA – immunofluorescent assay; EIA – enzyme immuno-assay.

**Figure 4:**
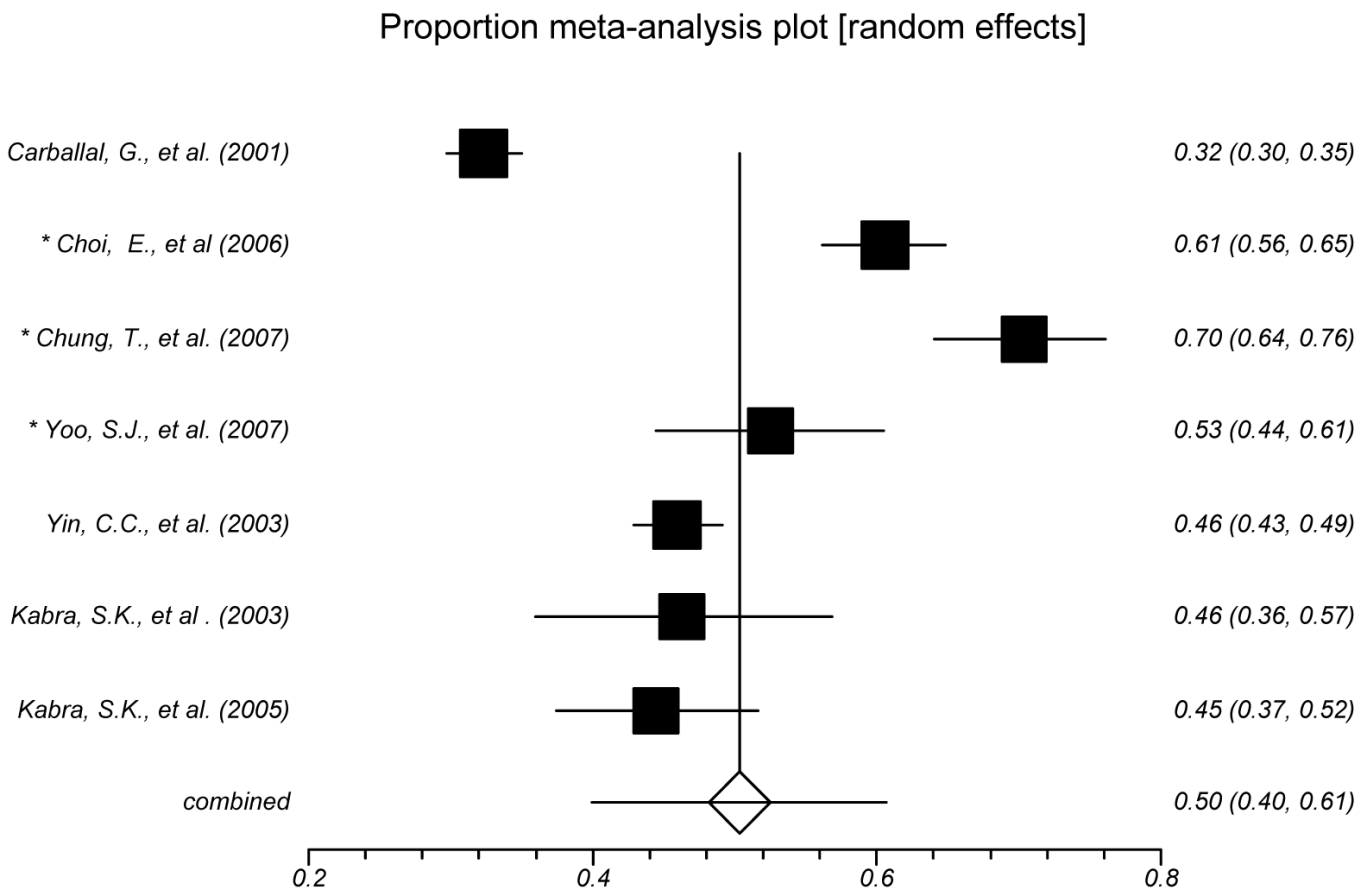
Meta-analysis of the proportion of patients aged 0-4 years with severe acute lower respiratory infections (ALRI) in whom a viral infection was detected (asterisk denotes investigation by polymerase-chain reaction).

Figure 4 presents the results of meta-analysis of the proportion of children with severe ALRI aged 0-4 years in whom at least one virus was detected (including RSV). Only studies that investigated three or more viruses were included in this analysis – 7 studies in total. This is an arbitrary cut off: these studies were deemed sufficiently active in their approach to detect viral infection, although the final result is likely to under-estimate the true burden. Pooled proportion was 50.4% (95% confidence interval [CI], 40.0% to 60.7%), with I˛ (inconsistency) parameter estimate of 97.0% (95% CI, 96.0% to 97.7%) (Table 2).

**Table 2:** Summary of estimated proportions of influenza virus (IV), parainfluenza virus (PIV), and adenovirus (AV) in all hospitalized acute lower respiratory infections (ALRI) and viral hospitalized ALRI using meta-analysis of eligible studies.

	Proportion (%)	95% confidence interval (%)
Proportion of all severe ALRI in children 0-4 y detecting:		
influenza	3.0	2.2-4.0
parainfluenza	2.7	1.9-3.7
adenovirus	5.9	3.4-9.1
Proportion of children with severe ALRI aged 0-4 y in whom at least one virus was detected (including respiratory syncytial virus, RSV):	50.4	40.0-60.7
Proportion of children with severe bronchiolitis aged 0-4 y in whom at least one virus was detected (including RSV):	66.3	56.2-75.6
Proportion of children with severe pneumonia aged 0-4 y in whom at least one virus was detected (including RSV):	48.7	38.0-59.4
Proportion of viral severe ALRI in children 0-4 y detecting:		
influenza	7.0	5.5-8.7
parainfluenza	5.8	4.1-7.7
adenovirus	8.8	5.4-13.0

### Pneumonia and bronchiolitis

Bronchiolitis as a clinical diagnosis is useful in identifying children who can be presumed unlikely to benefit from antibiotics. Pneumonia (especially focal) is known to have a different spectrum of etiological agents and antibiotics are usually warranted. Six studies that differentiate between these conditions were analyzed separately. The proportion of viruses detected in the bronchiolitis analysis was 66.3% (95% CI, 56.2% to 75.6%) and in the pneumonia analysis 48.7% (95% CI, 38.0% to 59.4%) (Table 2).

### Mixed viral ALRI

**Figure 5:**
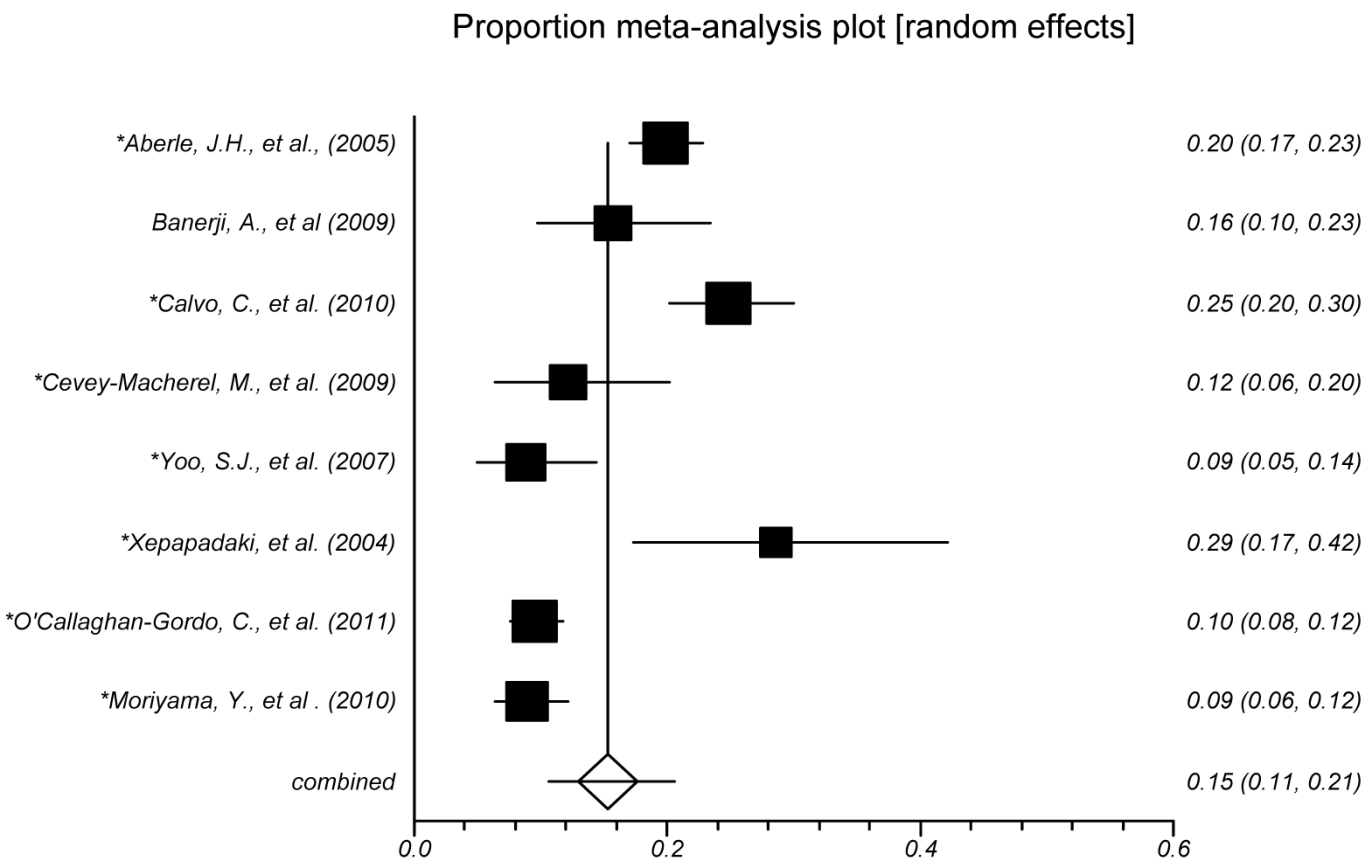
Meta-analysis of the proportion of patients aged 0-4 years with severe acute lower respiratory infections (ALRI) in whom multiple viral infections were detected (asterisk denotes investigation by polymerase-chain reaction).

Seven studies investigated three or more viruses using PCR method and reported the proportion of hospitalized childhood ALRI where mixed viral infections were detected (ie, more than one viral pathogen was confirmed). A meta-analysis of those studies showed that pooled proportion was 15.3% (95% CI, 10.6%-20.5%), with I˛ parameter estimate of 91.3% (95% CI, 85.7%-94.0%). Further analysis, which only included virus-positive ALRI cases, estimated that at least 21.9% (95% CI, 17.7%-26.4%) of the viral ALRI were mixed (Figure 5).

### Proportion of hospitalized ALRI due to influenza viruses

**Figure 6:**
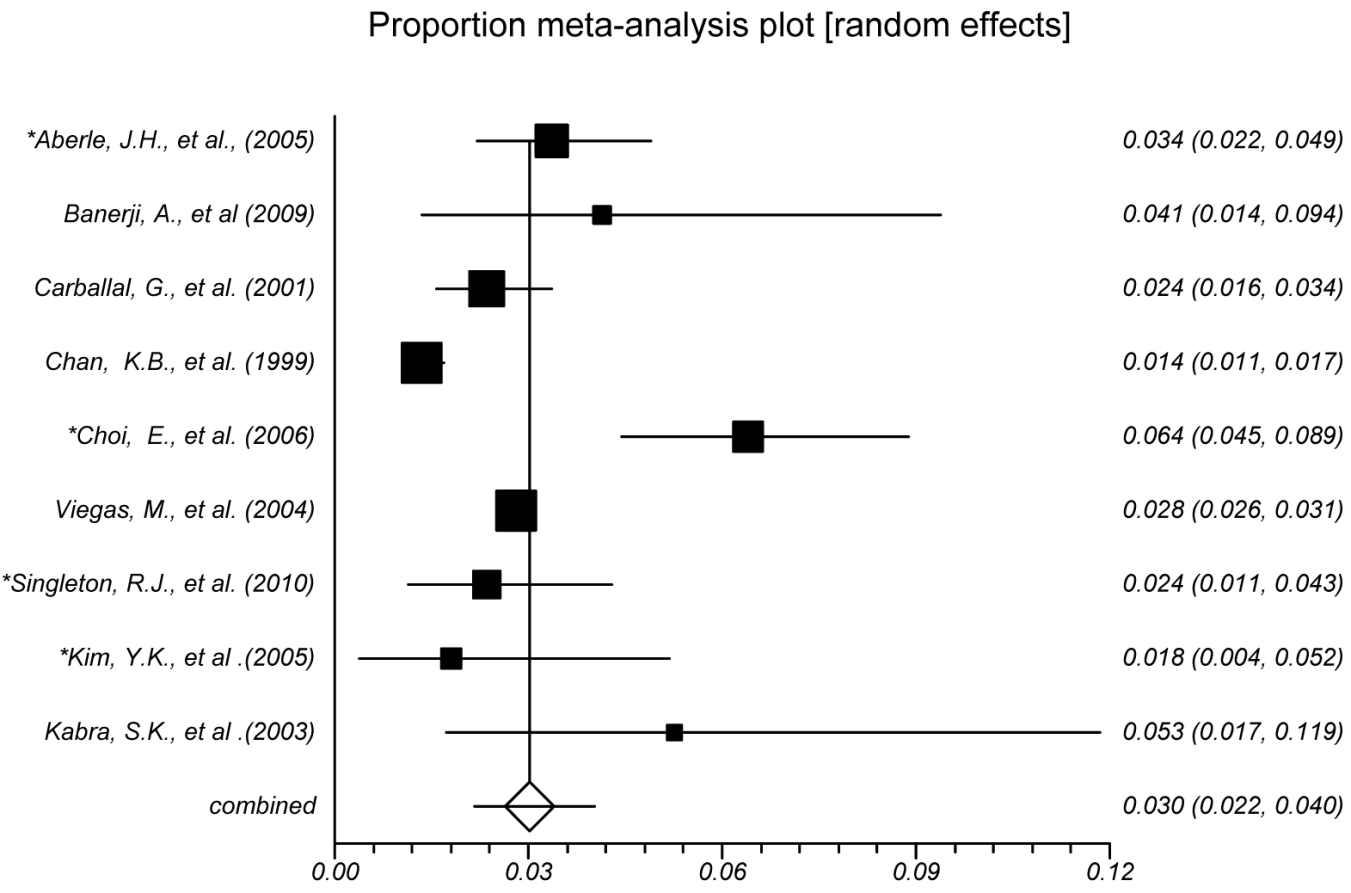
Meta-analysis of the proportion of patients aged 0-4 years with severe acute lower respiratory infections (ALRI) in whom a laboratory confirmed influenza infection was detected (asterisk denotes investigation by polymerase-chain reaction).

Meta-analysis included 9 studies in which IV infection was laboratory confirmed (Figure 6). We used only the 9 studies in which study population diagnosis was ALRI, rather than pneumonia or bronchiolitis separately. We estimated that 3.0% (95% CI, 2.2%-4.0%) of hospitalized ALRI in children were due to IV, with I˛ parameter estimate of 89.1% (95% CI, 81.7%-92.6%). Further analysis was performed to quantify IV infection as a proportion of all viral ALRI; IV accounted for 7.0% (95% CI, 5.5%-8.7%), with I˛ parameter estimate of 77.3% (95% CI, 47.2%-87.0%) (Table 2).

### Proportion of hospitalized ALRI due to parainfluenza viruses

**Figure 7:**
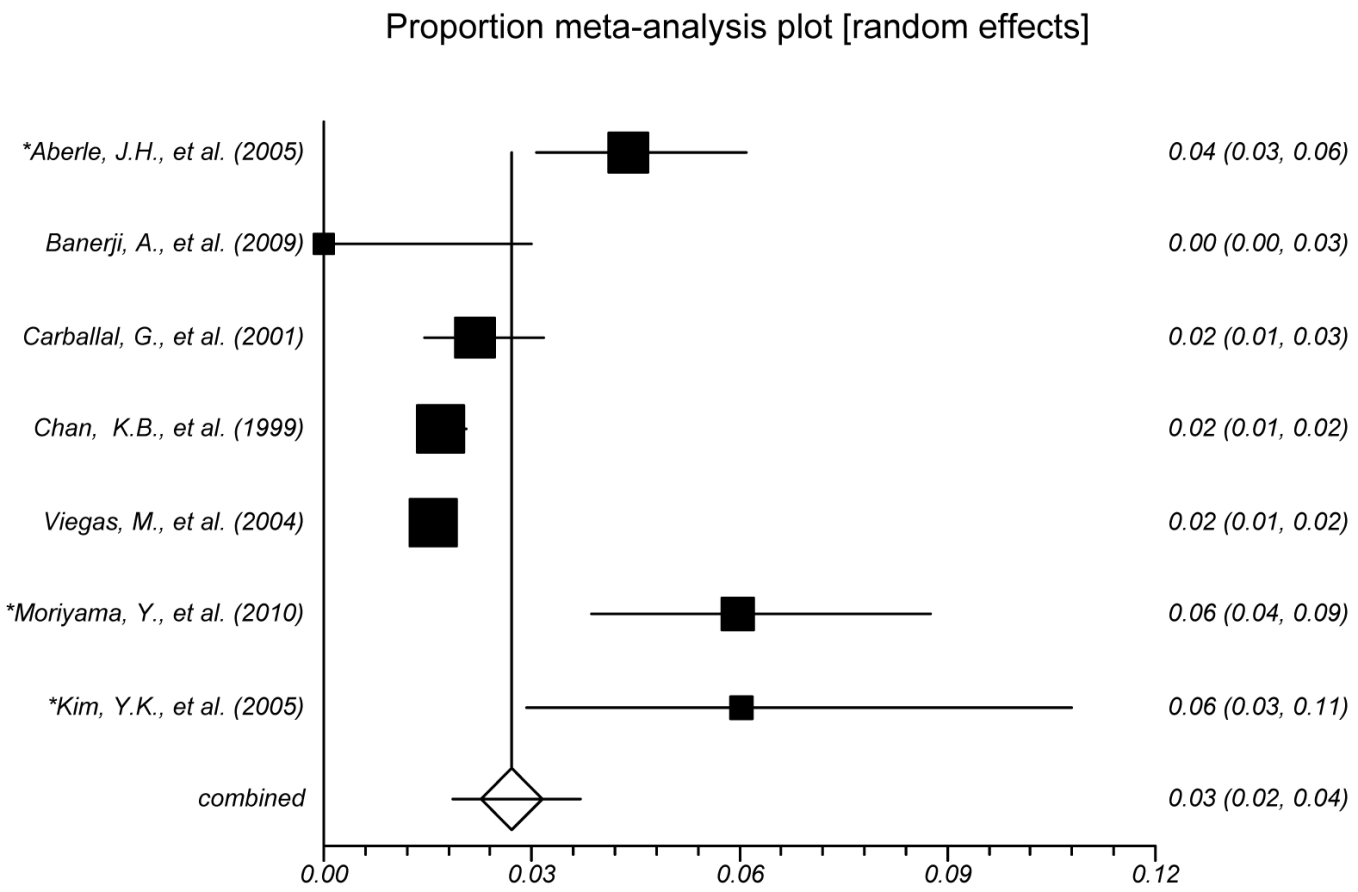
Meta-analysis of the proportion of patients aged 0-4 years with severe acute lower respiratory infections (ALRI) in whom parainfluenza infection was confirmed (asterisk denotes investigation by polymerase-chain reaction).

Meta-analysis included 7 studies in which PIV infection was confirmed (Figure 7). Similarly to IV infection, we only included those 7 studies where diagnosis was ALRI, rather than pneumonia or bronchiolitis separately. This analysis also excluded 3 studies where croup was a suspected diagnosis, because PIV are the major cause of croup. We estimated that 2.7% (95% CI, 1.9%-3.7%) of hospitalized ALRI in children were due to PIV, with I˛ parameter estimate of 90% (95% CI, 82.0%-93.5%). Among all virus-positive hospitalized ALRI cases, PIV accounted for 5.8% (95% CI, 4.1%-7.7%), with I˛ parameter estimate of 85.4% (95% CI, 67.1%-91.5%) (Table 2).

### Proportion of hospitalized ALRI due to adenoviruses

**Figure 8:**
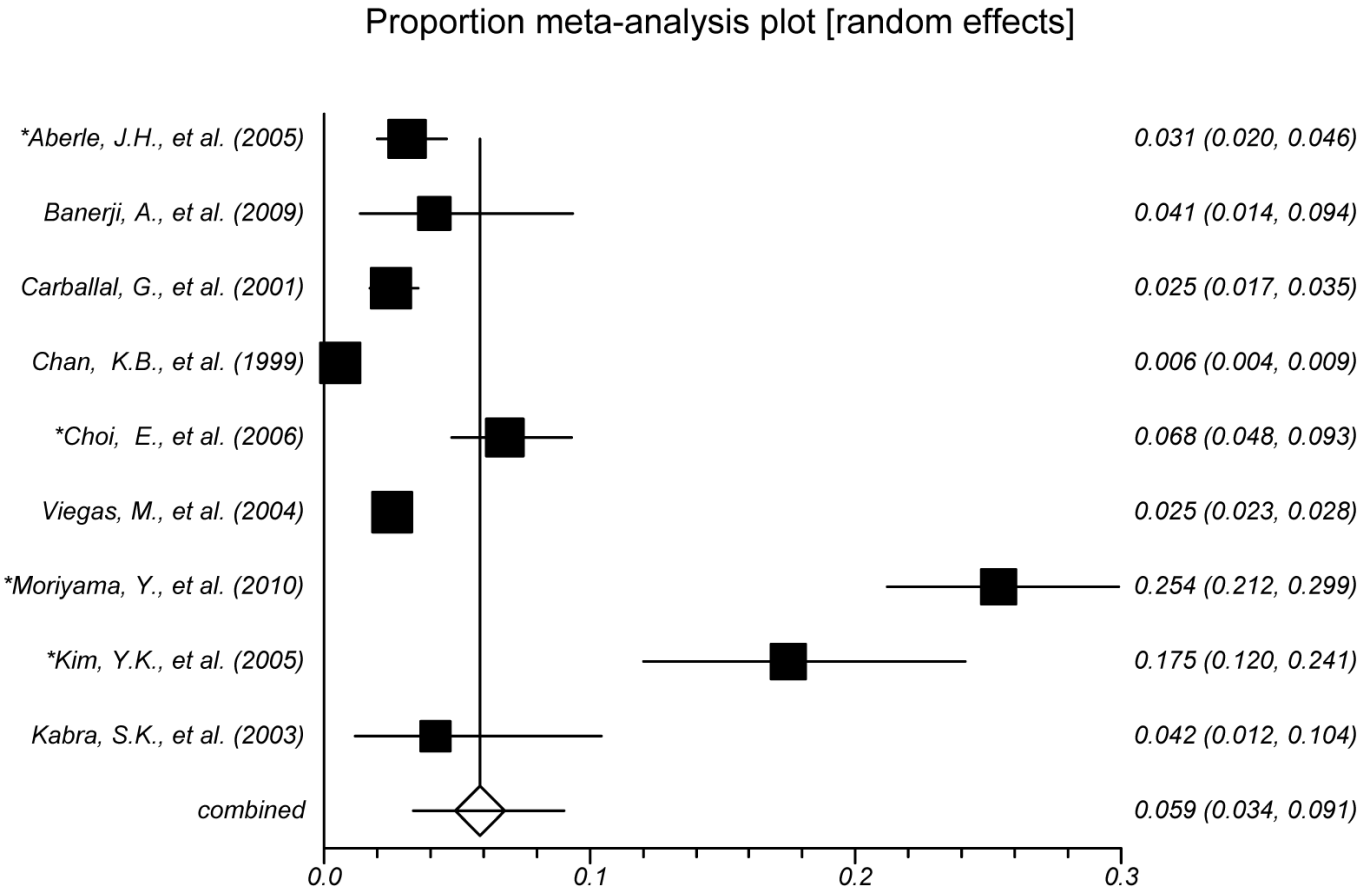
Meta-analysis of the proportion of patients aged 0-4 years with severe acute lower respiratory infections (ALRI) in whom adenovirus infection was confirmed (asterisk denotes investigation by polymerase-chain reaction).

Meta-analysis included 9 studies in which AV infection was confirmed (Figure 8). Similarly to IV infection, we only included those 9 studies in which diagnosis was ALRI, rather than pneumonia or bronchiolitis separately. We estimated that 5.8% (95% CI, 3.4%-9.1%) of hospitalized ALRI in children were due to AV, with I˛ parameter estimate of 98.2% (95% CI, 97.8%-98.5%). Among all virus-positive ALRI cases, AV accounted for 8.8% (95% CI, 5.3%-13.0%), with I˛ parameter estimate of 96.3% (95% CI, 94.9%-97.1%) (Table 2).

### Proportion of hospitalized ALRI due to coronaviruses

The number of available studies on human CV was much smaller than on the other 3 viruses: 8 studies were retained after the initial review, but they did not provide sufficient epidemiological information on the role of CV to perform the meta-analysis and develop reliable estimates. CV are technically difficult to culture, and they were the sole virus detected in 4.8% of patients in one ALRI study (94) and were detected in 10.6% of virus-positive pneumonia patients in another study (59) (supplementary Figure S1(supplementary Figure 1)).

## Discussion

This review estimated that 50% of all hospitalized ALRI in pre-school children, 66% of hospitalized bronciolitis episodes, and 49% of hospitalized pneumonia episodes showed viral involvement. All these estimates were derived using very heterogeneous sets of studies (I^2^>90% in many analyses). This heterogeneity is not surprising, given the large differences in study methods used, participants’ ethnicity, climate and viral endemicity, to name a few. For this reason, random effects models were used, sacrificing statistical power to ensure estimates that would be as valid as realistically possible with available information.

In one third of the episodes of bronchiolitis in hospitalized patients, no virus could be detected, although bronchiolitis is expected to be almost exclusively of viral etiology. We could hypothesize that this lack of sensitivity could be attributed both to imperfections of the tests and the timing of obtaining the sample. It is also possible that, in some studies, the diagnostic process for bronchiolitis can include some asthmatic (non-viral) patients (12). If we assume that the etiological estimates for pneumonia are subject to the same lack of sensitivity, this would mean that our estimates are likely to present a lower bound of the true role of viruses in all ALRI, and that the likely direction of bias is toward under-estimation of the true burden of viruses.

Detection of viral etiologies in hospitalized ALRI has been markedly increased by the use of PCR. We estimated that multiple viruses were involved in at least 15.3% of all cases of ALRI and 21.9% of virus positive ALRI cases. Both of those figures are likely to be underestimates, because they were based on studies that only tested for a limited number of viruses, and not for all known viruses (96).

The analyzed studies might have been affected by differences in regional practice: there were subtle differences in defining criteria for bronchiolitis in different areas (12) and inter-observer reliability in assessing some clinical signs has been low (97). Furthermore, some regions are prone to classifying tracheobronchitis and croup as LRTIs. According to several textbooks of respiratory and pediatric medicine these should be considered upper respiratory tract infections (URTIs) (5,6), because the vocal cords are not the division between upper and lower airway. Surprisingly, no study included in this review detailed the study populations’ past vaccinations; in areas where vaccination against bacterial causes has been implemented, higher proportion of viral etiology would be expected. It also seems likely that a higher proportion of viral ALRI cases that are complicated by bacterial infection would be hospitalized than of those that are pure viral infections. It was beyond the scope of this study to consider virus seasonality or age breakdown for specific viral infections. In contrast to the other main respiratory viruses, PIV has been suggested to cause ALRI more frequently in summer months, while IV is thought to affect older children than RSV (3). Further work is necessary to elucidate these issues.

The delay between sample collection and establishing a diagnosis makes identifying causative agents of little practical use in the majority of acute cases of ALRI. Thus, good etiological epidemiology is important to guide management. There is an argument, both economic and humanitarian, for prevention of viral ALRI over cure. Antiviral agents (with the possible exception of neuraminidase inhibitors for IV) have been shown largely ineffective. Following the successes of vaccination programs for bacterial ALRI, this review makes the case for the growing importance of viral agents and provides the first comprehensive estimates for the burden of viral etiology other than RSV.

Our study conveys some very broad and general messages for the further development of global health policy. First, there seems to be a viral etiological component to at least half of all severe ALRI that require hospitalization, and this number is probably an underestimate for the reasons discussed in this study. This is somewhat unexpected, because it establishes a larger role for viruses in severe ALRI than generally presumed in international health community. Second, although RSV is a dominant viral cause, the role of influenza, parainfluenza, adenoviruses, and coronaviruses should not be neglected: they seem to be jointly responsible for at least a third of all viral severe ALRI and one in six of all severe ALRI. Given that respiratory viruses are amenable to prevention through vaccination, and that their role at the community level is likely to be larger than at the hospital level, our study should allow for modeling of cost-effectiveness of developing such vaccines. With a global roll-out of the existing vaccines against *S. pneumoniae* and *H. influenzae*, viral etiology of ALRI will come under increased focus, and understanding the burden associated with particular viral pathogens should help plan global prevention and save further lives.
