# Supplementary material for: Viral etiology of hospitalized acute lower respiratory infections in children under 5 years of age – a systematic review and meta-analysis
Source: Croat Med J. 2013 Apr;54(2):122–34. doi: 10.3325/cmj.2013.54.122 (PMC3641872; doi:10.3325/cmj.2013.54.122)

SUPPLEMENTARY ONLINE MATERIAL – SEARCH STRATEGY

Medline (Ovid)

Studies that describe epidemiology in severe cases.

Viruses

1. exp respiratory syncytial viruses/ or respiratory syncytial virus, human/
2. Respirovirus Infections/
3. RSV.mp.
4. Respiratory Sync?tial Virus.mp.
5. 1 or 2 or 3 or 4
6. exp Orthomyxoviridae/
7. exp Influenza, Human/
8. (Influenza virus$ or Influenza$).mp.
9. 6 or 7 or 8
10. exp Paramyxoviridae Infections/
11. PIV.mp.
12. exp Paramyxoviridae/
13. Parainfluenza.mp.
14. 9 or 10 or 11 or 12
15. exp Adenoviridae/
16. exp Adenoviridae Infections/ or Adenovirus Infections, Human/
17. (Adenovirus$ or Adenovirus Infection$).mp.
18. 15 or 16 or 17
19. exp Metapneumovirus/
20. hMPV.mp.
21. human metapneumovirus.mp.
22. (MPV$ or MPV infection$ or metapneumovirus infection$).mp.
23. 19 or 20 or 21 or 22
24. exp Rhinovirus/
25. Rhinovirus$.mp.
26. Rhinovirus Infection$.mp.
27. 24 or 25 or 26
28. parvoviridae/ or parvovirinae/ or exp bocavirus/ or exp human bocavirus/ or Parvoviridae Infections/
29. (HBoV or Bocavirus).mp.
30. 28 or 29
31. coronaviridae/ or exp coronavirus/
32. exp Coronavirus Infections/
33. (CoV or SARS or Severe Acute Respiratory Syndrome Virus).mp.
34. coronavirus.mp.
35. 31 or 32 or 33 or 34
36. 5 or 9 or 14 or 18 or 24 or 27 or 30 or 36

ALRI

37. exp pneumonia/ or exp pneumonia, viral/
38. acute respiratory illness$.mp.
39. exp Respiratory Tract Infections/ or exp Respiratory Tract Diseases/
40. (ALRI or LRI or LRTI).mp.
41. bronchiolitis.mp. or exp Bronchiolitis/ or exp Bronchiolitis, Viral/
42. 37 or 38 or 39 or 40 or 41
43. 36 and 42

44. limit 43 to (humans and yr="1995 -Current" and ("all infant (birth to 23 months)" or "newborn infant (birth to 1 month)" or "infant (1 to 23 months)" or "preschool child (2 to 5 years)"))

Results: 6255
[mp=protocol supplementary concept, rare disease supplementary concept, title, original title, abstract, name of substance word, subject heading word, unique identifier]

Embase (Ovid)

Viruses

1. exp Respiratory syncytial pneumovirus/
2. (RSV or Respiratory syncytial virus).mp.
3. 1 or 2
4. exp influenza/ or exp orthomyxovirus infection/
5. influenza.mp.
6. 4 or 5
7. exp Parainfluenza virus infection/
8. (PIV or Paraflu or Parainfluenza).mp.
9. 7 or 8
10. exp Human adenovirus/ or exp Adenovirus/ or Adenovirus.mp. or exp adenovirus infection/ or exp human adenovirus infection/
11. Metapneumovirus infections.mp. or exp Metapneumovirus infection/ or exp Metapneumovirus/
12. exp human metapneumovirus/ or exp human metapneumovirus infection/
13. (hMPV or metapneumovirus).mp.
14. 11 or 12 or 13
15. exp Rhinovirus/ or Rhinovirus.mp. or exp Human rhinovirus/ or exp Rhinovirus infection/
16. exp Bocavirus/ or exp Bocavirus infection/ or exp human bocavirus/
17. HBoV.mp.
18. 16 or 17
19. Coronavirus.mp. or exp Coronavirus infection/ or exp Coronavirus/ or exp Human coronavirus NL63/
20. (CoV or SARS).mp.
21. 19 or 20
22. 8 or 11 or 14 or 15 or 19 or 20 or 23 or 26

Etiology

23. exp etiology/ or exp virus etiology/
24. (aetiology or etiology or cause$ or causative).mp.
25. 23 or 24

ALRI

26. exp bronchiolitis/ or exp respiratory tract infection/ or exp lower respiratory tract infection/ or exp pneumonia/
27. (ALRI or LRI or LRTI or acute respiratory illness$ or bronchiolitis or pneumonia).mp.

28. virus infection/ or exp viral bronchiolitis/ or exp virus pneumonia/ or exp viral respiratory tract infection/
29. 26 or 27 or 28
30. 22 and 25 and 29
31. limit 35 to (human and yr="1995 -Current" and (infant or child or preschool child <1 to 6 years>)) [mp=title, abstract, subject headings, heading word, drug trade name, original title, device manufacturer, drug manufacturer, device trade name, keyword]

Results: 2048

Global Health

Prevalence terms

1. exp disease prevalence/ or exp disease distribution/ or exp diseases/ or exp epidemiology/ or exp outbreaks/ or exp seroprevalence/
2. exp patients/
3. exp hospitals/ or exp intensive care units/ or hospital care/
4. exp disease statistics/ or exp statistical data/ or exp epidemiology/
5. (proportion or population or population-based or hospital-based or hospital$).mp.
6. 1 or 2 or 3 or 4 or 5

Viruses

7. exp human respiratory syncytial virus/ or exp pneumovirus/
8. (Respiratory Syncitial Virus$ or RSV or Respiratory Sync$tial virus$ or RSV infections or Respiratory Syncytial Virus Infections).mp.
9. 7 or 8
10. exp influenza/ or Influenza.mp.
11. exp influenza/ or exp influenza viruses/ or Influenza.mp.
12. (Influenza virus$ or flu or influenza).mp.
13. 10 or 11 or 12
14. exp parainfluenza viruses/ or exp parainfluenza/ or parainfluenza.mp.
15. (PIV or Paraflu or Parainfluenza or Paramyxoviridae).mp.
16. 14 or 15
17. Adenovirus.mp. or exp Adenoviridae/
18. (Adenovirus infections or Adenovirus$).mp.
19. 17 or 18
20. exp Human metapneumovirus/ or metapneumovirus.mp. or exp Metapneumovirus/
21. (Metapneumovirus infections or metapneumovirus).mp.
22. (hMPV or MPV).mp.
23. 20 or 21 or 22
24. exp rhinovirus/ or exp human rhinovirus a/ or exp human rhinovirus b/ or exp human rhinoviruses/
25. (Rhinovirus$ or Rhinovirus Infections$).mp.
26. 24 or 25
27. exp Human bocavirus/ or exp Bocavirus/ or Bocavirus.mp.
28. (HBoV or Human Bocavirus$ or Bocavirus$).mp.
29. 27 or 28
30. Coronavirus.mp. or exp Coronavirus/
31. (CoV or SARS or Coronavirus$ or Coronaviridae).mp.
32. 30 or 31
33. 9 or 13 or 16 or 19 or 23 or 26 or 29 or 32

Aetiology

34. exp aetiology/ or exp mixed infections/
35. (etiology or aetiology or cause$ or causative).mp.
36. 34 or 35

ALRI

37. exp community acquired pneumonia/ or exp pneumonia/
38. exp lower respiratory tract infections/ or exp respiratory diseases/
39. exp bronchiolitis/
40. (bronchiolitis or ALRI or LRI or LRTI or acute respiratory illness$).mp.
41. 37 or 38 or 39 or 40
42. 6 and 33 and 36 and 41
43. limit 42 to yr="1995 -Current"

[mp=abstract, title, original title, broad terms, heading words]

Results for All Searches

Searches completed on 12th August:

Results:

Medline: 6255
Embase: 2084
Global Health: 1295
CINAHL: 1634
AIM: 10
LILACS: 821
IMEMR: 169
IMSEAR:299
SciElo: 160
WHOLis: 42
WPRIM: 290

905 references discarded from Global Health as outside study date range.

Figure S1: An assessment of research interest in human Corona viruses, measured by number of publications and citations per year between 2000-2010 in ISI Web of Knowledge (search performed on 15 November 2011).

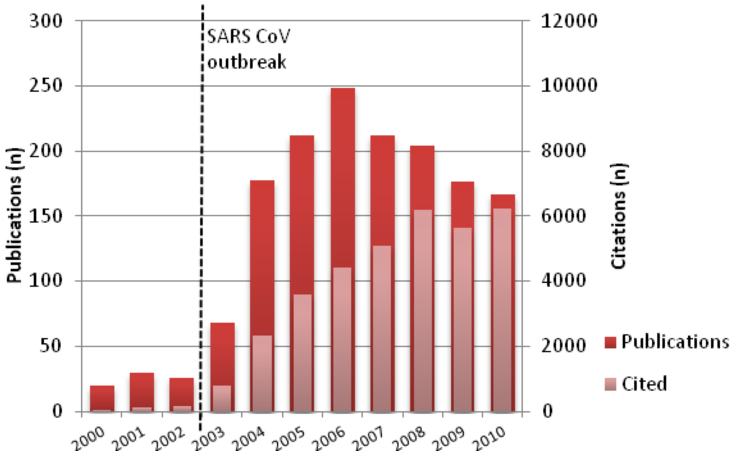

Supplement: Supplementary material [file CroatMedJ_54_s007.pdf]
