# Supplementary figures and images for: Viral etiology of hospitalized acute lower respiratory infections in children under 5 years of age – a systematic review and meta-analysis
Source: Croat Med J. 2013 Apr;54(2):122–34. doi: 10.3325/cmj.2013.54.122 (PMC3641872; doi:10.3325/cmj.2013.54.122)

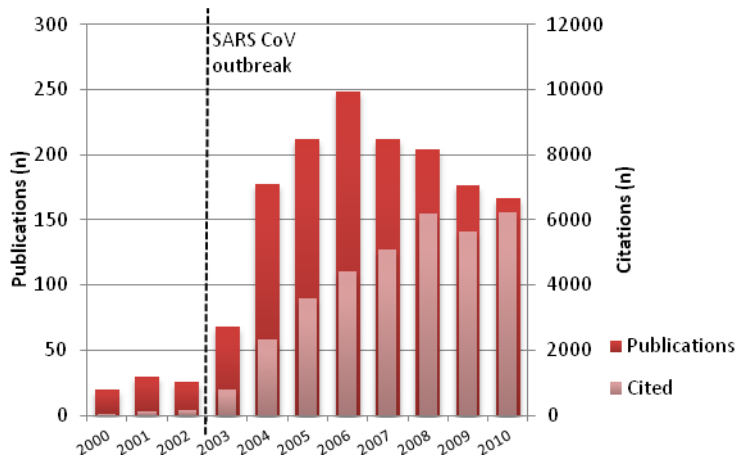

Supplement: Supplementary Figure 1 [file CroatMedJ_54_s008.pdf]
